# Navigating Ternary Doping in Li‐ion Cathodes With Closed‐Loop Multi‐Objective Bayesian Optimization

**DOI:** 10.1002/adma.202519790

**Published:** 2026-02-12

**Authors:** Nooshin Zeinali Galabi, Cheng‐Hao Liu, Moksh Jain, Marc Kamel, Shipeng Jia, Yoshua Bengio, Eric McCalla

**Affiliations:** ^1^ McGill University Montreal Quebec Canada; ^2^ Mila‐Quebec AI Institute Montreal Quebec Canada; ^3^ Université de Montréal Montreal Quebec Canada

**Keywords:** closed‐loop material design, high‐throughput experimentation, li‐ion battery cathodes, machine‐learning, ternary doping

## Abstract

To further improve secondary battery materials, we are increasingly exploring highly complex composition spaces in attempts to optimize multiple properties simultaneously. While our past work has done this in systematic manners using high‐throughput experimentation, the exponential increase in the search space with triple doping makes grid search prohibitively expensive. Here, we demonstrate a closed‐loop, multi‐objective machine learning approach to guide the high‐throughput workflow to efficiently navigate a space with approximately 14 million unique combinations. The test system is LiCoPO_4_, which we have previously explored using systematic codoping that was effective in optimizing one property only: energy density. To learn multiple electrochemical metrics, we first pretrain a set transformer on the public Materials Project database as a feature extractor, then attach a multi‐task Gaussian process head and finetune the entire model on our high‐throughput data. Through 3 rounds of active learning, we demonstrate that with a very small number of samples (as few as 125 random compositions and 63 predicted), we are able to simultaneously optimize four key electrochemical properties. Relative to the undoped system, the best composition raises our composite figure of merit up to five times. This establishes an end‐to‐end workflow for accelerated battery materials design to be used in the rapidly growing field of autonomous materials discovery.

## Graphical abstract

The search for advanced battery materials is pushing us into highly complex composition spaces. Here, a space with about 14 million unique combinations is efficiently explored using high‐throughput experimentation guided by Bayesian optimization with a deep kernel trained on both the Materials Project database and our data. The outcome is a cathode material optimized for four important battery metrics.

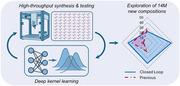

## 1 Introduction

Advanced rechargeable batteries have been the subject of extensive research over the past 40+ years. This has led to revolutionary innovations, ultimately giving us state‐of‐the‐art Li‐ion batteries which can both store a great deal of energy and have an impressive lifetime, enabling commercialized electric vehicles. Some key innovations were made at the material level, and this has resulted in a significant increase in the complexity of the materials. For example, the first commercial cathode was LiCoO_2_, which has ultimately been replaced by far more complex materials such as ceramic‐coated single‐crystal LiNi_1‐x‐y_Co_x_Mn_y_O_2_ [1, 2]. Dopants are also often used to further improve the material's performance [3]. This means that vast composition spaces are being explored to identify optimal next‐generation cathodes.

It is also becoming evident that despite impressive past developments in battery materials, there is a great deal of work left to meet the stringent demands imposed by daunting applications such as full electrification of transportation and grid storage [4, 5]. In such large‐scale applications, it is imperative to develop new battery chemistries with a particular emphasis on more diversified compositions to avoid potential supply limitations [6, 7, 8, 9]. Several methodologies are currently being developed to expand the composition spaces that can be explored while designing new battery materials. The McCalla lab has established high‐throughput experimental workflows to study all key components of Li‐ion, Na‐ion, and all‐solid batteries [10, 11, 12, 13, 14, 15]. To date, these methods have been utilized via systematic composition screening (e.g., preparing samples evenly spread throughout a Gibbs triangle [16, 17, 18] or doping a particular material with the same substitution level for more than 45 different dopants [19, 20, 21]). While highly informative, such approaches are inherently limited in terms of composition complexity. As efforts continue toward so‐called high entropy battery materials, there are no serious efforts to date to thoroughly screen the high entropy spaces, rather authors make a few compositions in a space with many elements and comment on performance. Even these limited explorations are yielding beyond state‐of‐the‐art performance for battery materials [13]. This raises obvious questions, such as what is the ceiling in terms of performance? Can broader explorations be used to achieve such optimal battery materials?

There is, therefore, an increasing need to begin to explore higher‐order phase spaces in a more comprehensive manner. In our past work, we systematically doped LiCoPO_4_ to improve both the energy density and the extended cycling [19]. The approach was to test 47 different dopants on their own. Upon finding that indium was highly effective in increasing the energy density (via improvement in ionic conductivity), we then codoped In‐containing materials to find an optimal In‐Mo codoped material that showed a balance of high capacity of 160 mAh/g very near the theoretical of 167 mAh/g, and capacity retention during cycling (76% over ten cycles). Both metrics are dramatically improved over those of the undoped, which showed a capacity of about 100 mAh/g and 50 % retention over ten cycles. Other materials showed a 100% retention over the ten cycles, but with considerably lower capacity. No material was able to fully optimize both of these metrics simultaneously [19]. Other challenges with LCP are that the irreversible capacity can be quite elevated and the overpotential can also be very high [22]. We made no efforts to optimize those two properties in our past work.

The concept of ‘self‐driving’ laboratories with model‐driven screening has recently emerged as a workflow for accelerating materials discovery [23, 24, 25]. In this context, Bayesian optimisation (BO) supplies a statistically principled active‐learning loop, especially in a low‐data regime, in which a surrogate model with quantified uncertainty is used to propose new experiments by maximising the experiments’ utility [26]. In recent years, BO has been applied in reaction optimization [27, 28, 29, 30], material synthesis [23], and more [31]. Yet, classical Gaussian processes (GPs) used in BO struggle in representing sparse, high‐dimensional, categorical–continuous inputs even with special treatment/embeddings [32, 33], and this is typical in battery compositions where complex battery chemistries are often described solely by elemental fractions. Deep‐kernel learning (DKL), on the other hand, remedies this by coupling a neural feature extractor with a GP, thereby retaining Bayesian calibration while capturing complex, non‐linear interactions from latent representations [34, 35, 36, 37].

**FIGURE 1:**
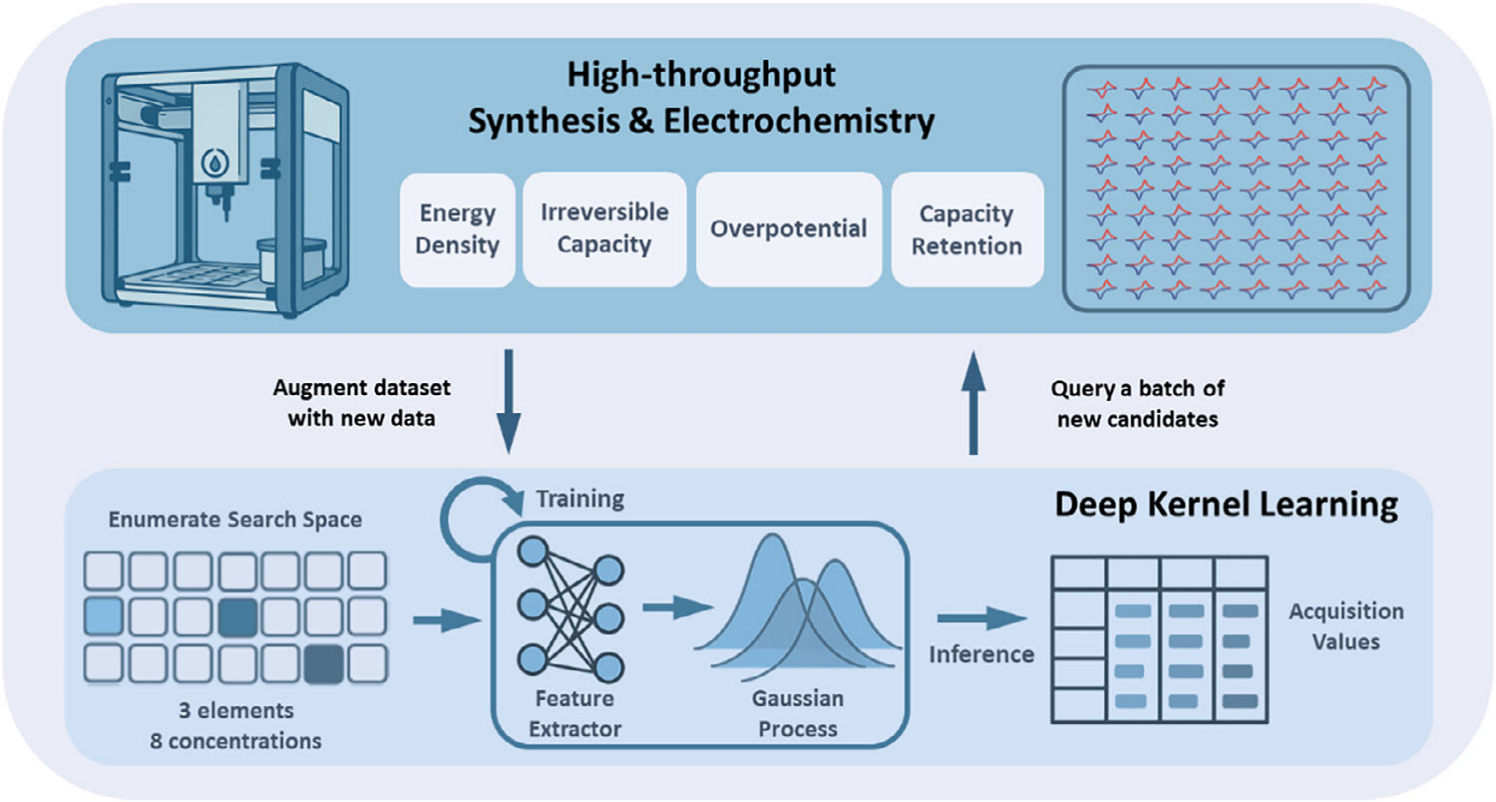
Workflow used in this project, where experimental electrochemical data is supplemented and guided by a ML loop.

Herein, we use LCP as a test system to demonstrate an end‐to‐end, closed‐loop workflow for battery material compositions design that couples the high‐throughput workflow in the McCalla lab (Figure 1). Specifically, we aim to simultaneously optimize all four performance metrics mentioned above (energy density, capacity retention, irreversible capacity, and overpotential); though the ML method is developed to optimize any number of properties simultaneously, and in arbitrary host materials. The approach is to explore a highly complex composition space: triple doping with dopants selected from a list of 56 dopants that can be introduced at 8 different doping levels. Importantly, the only input to the ML workflow was battery performance metrics, with no inputs related to structure or phase purity, the objective is to develop the materials purely based on battery performance. Remarkably, this works very effectively with a relatively small number of samples, such that this work will guide further efforts in self‐driving labs aiming to make better batteries.

## 2 Experimental Methods

### 2.1 Deep‐Kernel Surrogate

To represent dopant concentrations, each candidate is specified by an unordered set of three‐element concentration pairs $C={\left\{\left(e_{i},c_{i}\right)\right\}}_{i=1}^{3}\;$, where *e_i_*, is an element symbol and $c_{i}\in \left(0,0.10\right]$ is its composition fraction relative to Co. Elements are represented with a one‐hot encoding. Concentrations are normalized and appended as scalars. To model the four property responses of triple‐doped LiCoPO_4_, we trained a deep‐kernel surrogate model. Doping element compositions can significantly affect the crystal structure, defect chemistry, phase purity, and morphology, which dominate battery performances but are difficult to predict a priori; therefore, we deliberately focus on capturing the effects of composition *sans* any structural information [38]. We use a set transformer encoder, ϕ_θ_(·) that produces permutation‐invariant descriptors for unordered element–concentration pairs. This flattened descriptor becomes the input of an exact Gaussian process with a Matérn kernel (ν = 5/2) and automatic relevance determination, i.e., **z** = vec(ϕ_θ_(**X**)). The Gaussian process head is multi‐task and can predict different experimental targets. To train the surrogate, we follow a two‐stage training. The set transformer is pretrained by regressing on DFT‐computed Fermi levels of ∼10^5^ inorganic compounds (filtered to compositions with ≤ 5 elements) from the Materials Project using the Adam optimizer with a learning rate of 5 × 10^−4^. This data represents diverse inorganic chemistries, and it allows the encoder to learn generalizable elemental representations that are indicative of electronic structure beyond the specific stoichiometry of the target system. Subsequently, to tailor to the dopant‐specific representation of the LCP system, we finetune the set transformer and train the GP by exact marginal likelihood using the Adam optimizer on the normalized data of 222 samples of one or two‐dopant data from our previous project, as well as on 125 samples of tri‐dopant data with random compositions. The learning rate is 5 × 10^−3^ with weight decay of 10^−4^, and we use employ an early‐stopping criterion. For model evaluation, we employed 5‐fold cross‐validation with an 85:15 train:test split.

### 2.2 Bayesian Optimization

With 56 permissible dopants and 8 concentration levels, the total design space contains $\left(\begin{array}{c} 56\\ 3 \end{array}\right)\times 8^{3}=1.42\times 10^{7}$ unique compositions. To select each round of candidates, we deterministically enumerated the full space and predicted the posterior mean μ(C) and standard deviation σ(C) in 512‐sample mini‐batches. We then evaluated the usefulness of each candidate by computing the multi‐objective upper confidence bound acquisition function $\alpha \left(C\right)=\sum_{j}w_{j}s_{j}\left(\mu_{j}\left(C\right)+\kappa \sigma_{j}\left(C\right)\right),\;\mathrm{where}\;\kappa =0.5$, and where w_j_ = +1 or − 1 for properties to maximize/minimize, respectively. Scaling *s_j_* rescales each metric to [0,1] to prevent dominance by any single range. This linear scalarization preserves Pareto ordering under a monotone transform and is cheap to evaluate. Inference of the light‐weight model is fast, with enumeration finishing within 20 min on a single L40S GPU. In each round, the 63 highest‐UCB unseen triplets were selected for synthesis and electrochemical testing, and their results are augmented.

### 2.3 Synthesis

LiCo_1‐x‐y‐z_D1_x_D2_y_D3_z_PO_4_ (D1, D2, and D3 are 3 dopants) materials were prepared in combinatorial batches of 64 samples using the sol‐gel method described in ref. [19]. In that study, we tested both 750°C and 850 °C sintering and generally found better performance for the higher temperature. Here, we only utilize 850 °C, but the approach detailed herein could easily be extended to also screen various temperatures and other synthesis condition parameters. We simply choose to focus on a highly complex composition space made under synthesis conditions that we know yield quality battery performance. Every batch prepared herein contained one undoped sample as a reference. The full list of dopants is provided in the full dataset. The doping levels, x,y, and z, could each take values from 0.01 to 0.08 in increments of 0.01. Two molar stock solutions of LiNO_3_, Co(NO_3_)_2_, (NH_4_)_2_HPO_4_, and 4 molar citric acid solution were used, with the concentration of the initial metal solutions quantified by inductively coupled plasma–optical emission spectroscopy (ICP–OES). The dispensing step was automated using the Opentrons Liquid Handler Pipetting Robot. First, the chelating agent, citric acid, was added in a 1:1 molar ratio (citric acid to the total metal cations) into 64 small alumina cups. Varying ratios of cobalt nitrate solution were then dispensed into cups according to the targeted doping level (sum of 3 dopants). Subsequently, the dopants were added using 0.3 m solutions, followed by the dispensing of lithium nitrate and ammonium phosphate dibasic solutions, respectively. The samples were dried at 80 °C overnight. To remove the remaining trapped water, incremental heating from 100 to 200 °C under vacuum was applied. Sintering was carried out in air at 850 °C for 4 h with a ramp/cool rate of 5 °C min^−1^. The resulting powder then was ground and mixed with citric acid solution (104 g L^−1^) for the carbon coating purpose with a ratio of 13 wt.% citric acid content (10 wt.% carbon), forming a slurry which then was spread in a thin layer on an alumina plate. They were then transferred to a tube furnace and heated to 650 °C for 30 min at a ramp rate of 2 °C min^−1^ under an Ar:H2 (95:5) atmosphere. The carbon‐coated samples were ground again for the structural and subsequently the electrochemical analysis.

### 2.4 Battery Performance

Electrochemical analysis was performed using a lab‐built high‐throughput cyclic voltammetry (CV) system designed to cycle 64 cathode materials simultaneously. To prepare the cathode electrodes, around 6 mg of each active material (AM) was weighed and mixed with a slurry containing 60 µL N‐methyl‐2‐pyrrolidone (NMP) (Alfa Aesar), 0.34 mg polyvinylidene fluoride (PVDF) (Kynar 1100), and 0.78 mg carbon black (TIMCAL). After mixing the slurry with a stir bar for about 15 to 20 min, 3 µL of this slurry was drop‐casted onto the aluminum pads on a custom‐designed printed circuit board (PCB, Optima Tech) with 64 parallel channels. The electrodes were then dried at 60 °C overnight. The combinatorial cell was then assembled in an argon‐filled glovebox using two Whatman microfiber separators soaked with electrolyte (1 M LiPF_6_ in 1:1 EC:DMC, SoulBrain MI), and a Li metal sheet as the counter electrode. The CVs were performed with the voltage range from 3.0 to 5.3 V vs. Li/Li^+^ using a sweep rate of 0.2 V h^−1^.

In order to effectively compare samples’ performances across batches (and across active learning cycles), we develop a new figure of merit (FOM) to quantify overall improvement in performance compared to the undoped material. $FOM\left(C\right)=\prod_{j\in M_{+}}\frac{x_{j}\left(C\right)}{x_{j}^{undoped}}\times \prod_{k\in M_{-}}\frac{x_{j}^{undoped}}{x_{k}\left(C\right)}$ with $M_{+}=$ energy density, retention, and $M_{-}=$ irreversible capacity and overpotential. Thus, as shown in Table 1, the FOM for an undoped material is by definition 1.0, while for our best material from ref. [19] we obtain 1.3. To give some perspective on this metric, an improvement of 50% on each battery property would give an FOM of 1.5^4^ = 5.06. This metric therefore, aims to weigh all 4 battery performance metrics equally. For statistical reporting, we define a ‘hit’ as any sample with all four metrics better than the undoped LCP. Lastly, to measure Pareto front exploration, we perform min‐max normalization of all data while capping outliers (retention capped at 150%, overpotential capped from 0.2 to 0.8V, irreversible capacity capped at 20‐200 mAh/g), and report the Pareto front size and the 4D hypervolume improvement via Monte Carlo sampling of 1000 samples.

**TABLE 1:** Extracted battery performance metrics for key materials in this study. The figure of merit (FOM) is calculated by taking ratios of each metric compared to the undoped value (the ratio is done to ensure >1 means improvement), and then we multiply all four ratios together to get the FOM. For example, for #3, the calculation is simply 660/511 × 73/42 × 82/70 × 0.713/0.431.

Sample	Dopant composition	Energy 1st cycle (Wh/kg)	Irreversible Capacity 1st cycle (mAh/g)	Capacity Retention after 5 cycles (%)	Overpotential 1st cycle (V)	FOM
**#1 ‐ Undoped**	—	511	73	70	0.713	1.000
**#2 ‐ Ref**. [19]	In_0.01_ Mo_0.01_	659	95	82	0.658	1.262
**#3 ‐ random, 1**st	Cr_0.03_ Mn_0.04_ Nd_0.02_	660	42	82	0.431	4.403
**#4 – random, 2**nd	Fe_0.05_ Ru_0.01_ Y_0.05_	616	42	69	0.513	2.926
**#5 ‐ random, 3**rd	Gd_0.02_ In_0.03_ Zr_0.02_	647	43	63	0.517	2.661
**#6 ‐ random, 4**th	P_0.04_ V_0.02_ Cd_0.05_	621	52	76	0.545	2.443
**#7 ‐ round 1, 1**st	Al_0.05_ Cs_0.01_ In_0.05_	626	39	98	0.445	5.145
**#8 – round 1, 2**nd	In_0.02_ Mn_0.03_ Nd_0.01_	610	59	88	0.486	2.712
**#9 – round 2, 1**st	Cu_0.01_ In_0.05_ Na_0.02_	701	46	85	0.474	4.004
**#10 – round 2, 2**nd	In_0.07_ Mn_0.07_ P_0.07_	666	56	94	0.410	3.992
**#11 – round 2, 3**rd	Cr_0.06_ In_0.06_ P_0.01_	612	54	93	0.411	3.768
**#12 – round 2, 4**th	Cr_0.01_ In_0.07_ Mn_0.07_	531	67	89	0.290	3.516
**#13 – round 3, 1**st	Ni_0.01_ P_0.02_ Sc_0.06_	655	49	81	0.467	3.406
**#14 – round 3, 2**nd	In_0.08_ Nb_0.01_ Pr_0.02_	598	37	78	0.553	3.295

### 2.5 Material Characterization

X‐ray diffraction was performed on the samples made in the last 2 rounds of active learning. Samples were characterized using high‐throughput powder X‐ray diffraction (XRD) conducted in transmission mode on a Panalytical diffractometer equipped with a molybdenum (Mo) anode X‐ray source operating at 60 kV and 40 mA and a GaliPIX3D area detector. Measurements were performed over a scattering angle range of 4–30° for Mo Kα radiation (λ = 0.70926 Å for Kα1), which is equivalent to the 10–70° range commonly used for copper (Cu) radiation. Each sample produced high‐intensity diffraction patterns, with peak counts exceeding 2000 within a 10‐min scan, suitable for detailed Rietveld refinement. The collected diffraction patterns were processed to remove Mo Kα2 contributions and converted to the Cu Kα1 scale (λ = 1.54051 Å) for consistency with literature standards. Phase identification and quantification were carried out using Panalytical's HighScore Plus software by searching against open‐access and ICSD databases. Subsequent Rietveld refinement was performed to determine the content of each phase.

## 3 Results and Discussion

**FIGURE 2:**
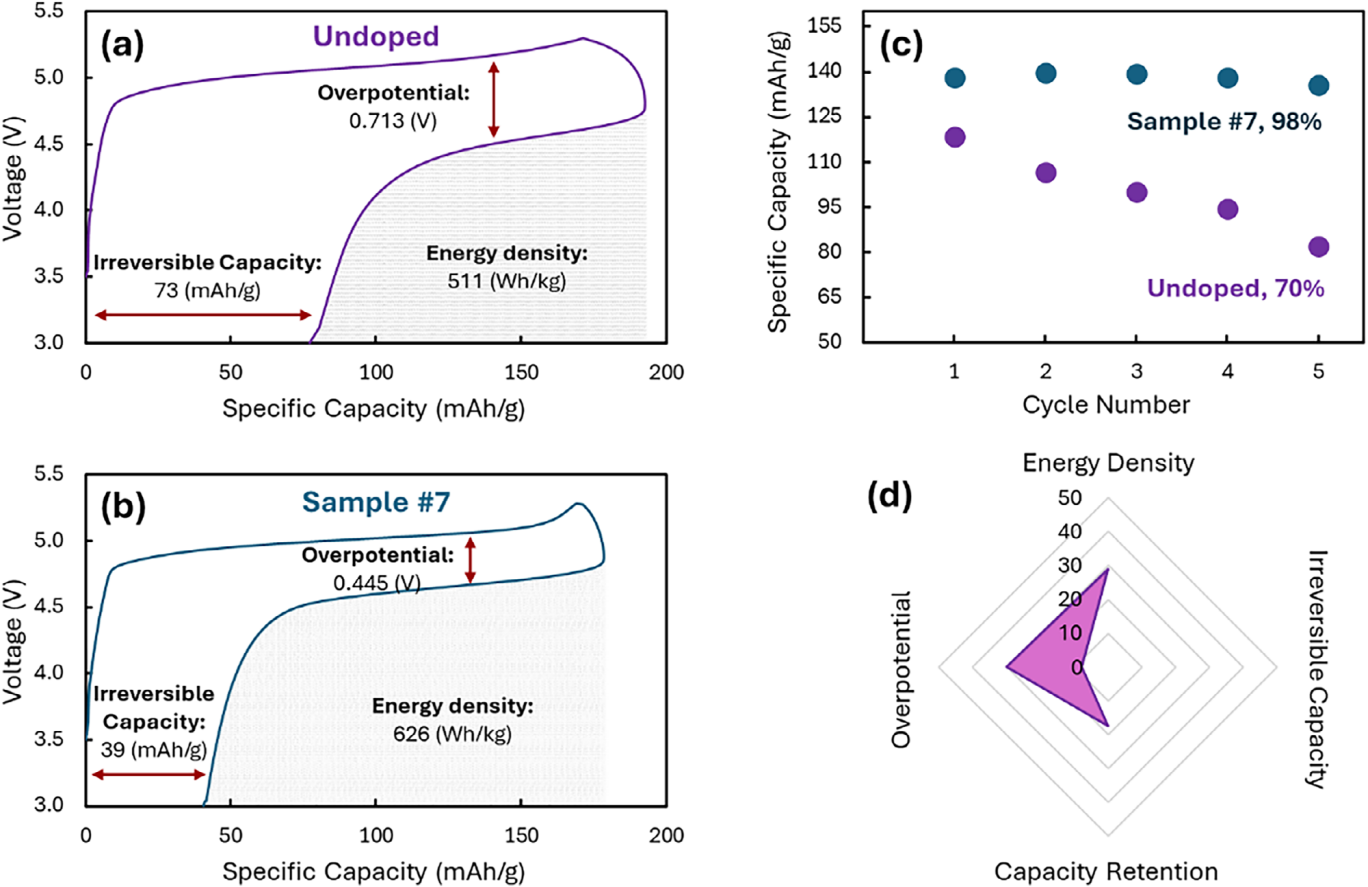
First cycle voltage curves for both the undoped sample (a) and sample #7 (b). The voltage curves illustrate how 3 of the 4 battery metrics are extracted from the data. The specific capacity over 5 cycles for both these materials (c) demonstrates how the 4th metric (capacity retention) is extracted. The radar plot for sample #2 (d) shows the percent improvement in each metric in comparison to the undoped material. Irreversible capacity shows a decreased performance compared to the undoped, and so it takes a negative value in the radar plot.

The four key battery metrics that we are trying to co‐optimize were extracted from 5 cycles of cyclic voltammetry as described in great detail elsewhere [10, 16, 19]. Figure 2a,b shows representative voltage curves extracted from the CVs for the first cycle. 3 of our battery metrics are directly extracted from these curves: the energy density is the area under the curve during discharge, the overpotential is the difference between the average charge and discharge voltages, and the irreversible capacity is the capacity left over at the end of the cycle (i.e., it is the difference between the capacity during charge and discharge). Figure 2b shows sample #7, which is a ML‐predicted composition that will be discussed in detail below. The figure clearly shows dramatic improvement on all 3 of these metrics as a result of the ML‐guided process herein. Figure 2c shows the capacity over 5 cycles for these two representative materials (undoped and #7), and these plots are used to extract the 4th battery metric, capacity retention (the discharge capacity from the 5th cycle divided by that of the 1st, expressed as a percentage). Again, sample #7 shows a very impressive improvement over the undoped. We can also express the level of improvement in all 4 metrics in a radar plot, as shown in Figure 2d. In this plot, each value is the improvement in the metric as compared to the value of the undoped material, expressed as a percentage. In the rare cases of the metric getting worse in our new samples, the value in the radar plot is negative, as shown for irreversible capacity in Figure 2d. By definition, the undoped material has a radar plot that is a single point at the origin (i.e., all metrics show no improvement over themselves). Again, the radar plot in Figure 2d clearly shows that our best material from ref. [19] improved 3 of the metrics, with energy density being particularly high, but in fact decreased the irreversible capacity. It is therefore the key objective of the current work to optimize all 4 metrics at once by exploring a composition space that is too complex to explore by traditional intuition‐based methods.

We then assess the ability of our deep‐kernel surrogate model to capture these complex, non‐linear dependencies among the battery performance metrics. We developed a modular and flexible Python package named BatteryBO based on GPyTorch, which can accommodate diverse chemical composition settings as well as future advances in composition featurization or GP. The relatively lightweight deep‐kernel surrogate combines a pretrained permutation‐invariant set transformer with an exact multitask Matérn‐ARD GP, yielding a fast non‐stationary kernel with closed‐form marginal likelihood over mixed categorical–continuous inputs that jointly learns multiple properties. This method bypasses the convergence challenges of Bayesian optimization in high‐dimensional spaces, as the set transformer encoder performs implicit dimensionality reduction, projecting variable‐sized compositional inputs to a fixed 8D latent representation, where the GP operates efficiently.

**FIGURE 3:**
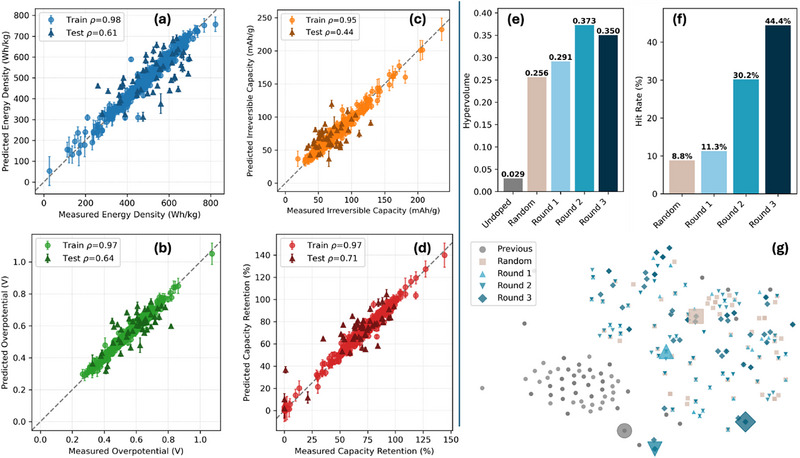
(a–d) Results of the final round of ML showing comparison of predicted and measured values for both the training and test sets. (e), (f) Hypervolume and hit rate analysis of each round, (g) t‐Distributed stochastic neighbour embedding (t‐SNE) of dopant compositions in each round of acquisition compared against the previous paper's data or random tri‐dopants experiments. The larger marker indicates the best sample in each dataset.

Figure 3a–d presents parity plots of predicted vs. experimental measurements for energy density, capacity retention, irreversible capacity, and overpotential, respectively. On the training set, both Spearman and Pearson correlations exceed 0.9; under random cross‐validation, Pearson correlation ranges from 0.48 to 0.85, and Spearman correlation is between 0.44 and 0.71, an expected drop that reflects the challenge of generalizing in a high‐dimensional, data‐scarce parameter space. Mean absolute errors remain between 6.5 and 10 % of each property's dynamic range. The model's predictive standard deviations correlate moderately with absolute errors (calibration slope range between 0.38 and 0.79), suggesting somewhat useful epistemic uncertainty quantification. To benchmark our model, we ablated against a simple one‐hot‐encoded GP, a multilayer perceptron featurizer with GP, a standalone set transformer, and a set transformer with GP but without pretraining. These baseline models deliver reduced test Pearson and Spearman correlations (Figures S10 and S11), which show that the pretrained, permutation‐invaraint feature embeddings provide a useful prior for further fine‐tuning when used in conjunction with GP. We note that while other BO methodologies/architectures are feasible, the current methods suffice for our application.

To evaluate whether the learned representations encode physical information, we related the latent embeddings to a set of 12 physically interpretable descriptors derived only from dopant information. Linear probe regression reveals that the latent space most strongly encodes configurational complexity, followed by ionic‐size effects and then weaker electronic structure proxies (Figure S12a). While individual correlations are moderate, canonical correlation analysis (CCA) further shows that the latent representation shares several low‐dimensional modes with the physical descriptor space: the first canonical component captures electronic and thermodynamic factors while the second is dominated by ionic size terms (Figure S12b,c). Finally, correlations between individual latent principal components and descriptors are generally modest (Figure S12d), indicating the embedding reflects multiple physical factors rather than any single descriptor.

To generate new candidates for experimental validation, the trained model is used to enumerate the entire search space with an acquisition function. To efficiently utilize parallel high‐throughput experiments, we sample in batches instead of performing typical sequential acquisition. To examine how our active learning approach balances exploration and exploitation, we visualized compositional embeddings from MatBERT via t‐SNE (Figure 3g) and UMAP (Figure S6) [39]. The model acquisition clearly exploits local clusters associated with prior paper/random training data, yet it can sometimes venture into previously unexplored regions.

The key data collected in this study are cyclic voltammograms (CVs) for 376 new materials, including both random compositions and predicted compositions across three rounds of active learning, all of which supplement our previous dataset of 474 samples from ref. [19]. In that work, the reproducibility of our battery metrics was carefully evaluated. Repeats of 1% In‐doped samples yielded a first‐discharge‐capacity RSD of 10.68%, representing an upper bound for batch‐to‐batch variability in our sol‐gel synthesis and electrochemical testing. In the present study, duplicate electrochemical cells across 64 samples gave an average RSD of 4.89%. Selected duplicate synthesis showed an average RSD of 4.7% for the 1st cycle discharge capacity. Voltage and irreversible capacity values typically show RSD values below 1% [10]. These results confirm that the variability introduced by our synthesis and battery testing workflow consistently falls within an acceptable range (10%). Figures S1 and S2 show representative first‐cycle voltage curves and the CVs they were calculated from. For simplicity, we label samples by the composition of the dopants only, i.e., Li[Co_1‐x‐y‐z_A_x_B_y_C_z_]PO_4_ is simply denoted A_x_B_y_C_z_ throughout this article. Figure S2 therefore clearly demonstrates that a number of the new materials (#3 onward) show important improvements over our past results (#1–2). Sample #7 is particularly stellar, as also highlighted in Figure 2. It shows a significant reduction in both overpotential (seen as a vertical offset between the charge and discharge voltage plateaus) and irreversible capacity, while also yielding a large area under the discharge curve.

However, visual determination of improvements is not feasible in such a multiobjective dataset, and we must rely on quantitative values of overall improvement. To do so, we calculate the FOM, hypervolume, and hit rate as described in the methods section. Unlike the acquisition function used to sample the new materials in the ML loop, the FOM only depends on the improvement compared to the undoped material and allows comparison between the various rounds of active learning. Meanwhile, hypervolume quantifies the exploration of the Pareto front, and hit rate shows whether every metric can outperform the undoped. Table 1 shows the materials with the largest FOM across each round of data collection. In the supporting dataset, we show this table with all data from this study; violin plots are provided in Figure S7.

Table 1 summarizes the highest‐FOM materials from each experimental round. Among 125 materials with random tri‐dopants, one achieved a very large FOM of 4.4 (in fact, it is the second best out of the whole table), but a very steep drop‐off follows for all remaining materials (2.9 and lower). We interpret this as our having been relatively lucky in the one highly performing random material; indeed, the hit rate for random dopants is only 8.8%. The first active learning round (63 new materials) yielded a best FOM of 5.1 with a significant drop off to 2.7 for the second‐ranked sample. By contrast, the 2nd round of active learning (126 new compositions) exhibited a more consistent performance, where the top 4 compositions were within 0.5 of each other, and the top performer still showed a very high FOM of 4.0. We interpret the slower drop off with the later rounds of predictions as being due to a lower dependence on chance as the dataset used in the predictions increases. The third, and final, round of active learning (63 new materials) achieved the highest hit rate, but the top performer showed a slight drop‐off in the largest FOM (3.4).

Aggregating across rounds, the average FOM of samples shows a strong improvement in the overall performance of the battery materials through ML‐driven design (Figure S9). The random tri‐doped samples showed a low average FOM of 1.05. Across successive prediction rounds, the average FOM increased progressively from 1.03 in the first round to 1.78 and ultimately reaching 2.13 in the final round. The average top‐decile FOM similarly improves from 2.39 ± 0.68 (the random tri‐dopant) to 2.73 ± 1.24, 3.34 ± 0.39, and 3.25 ± 0.09 across the three rounds, where the consistent reduction in variance indicates an improved identification of high‐performing compositions. The experimental hit rate also increases from 8.8% in random tri‐dopants to 44.4% in the last round of active learning (Figure 3f; Figure S9). The Pareto front size for each round follows the hit rate closely (7.2% for random, 8.1%, 16.7%, 47.6% for the three active learning rounds). To quantify the amount of Pareto exploration, the normalized hypervolume improvements reached up to 1168% relative to the undoped (Figure 3e), signifying substantial expansion of the accessible performance space across the four metrics. The Pareto‐depth distribution further indicates progressive coverage of deeper fronts (Figure S8). Together, these statistics show that the closed loop simultaneously optimizes multiple battery performances while expanding Pareto coverage round‐over‐round.

**FIGURE 4:**
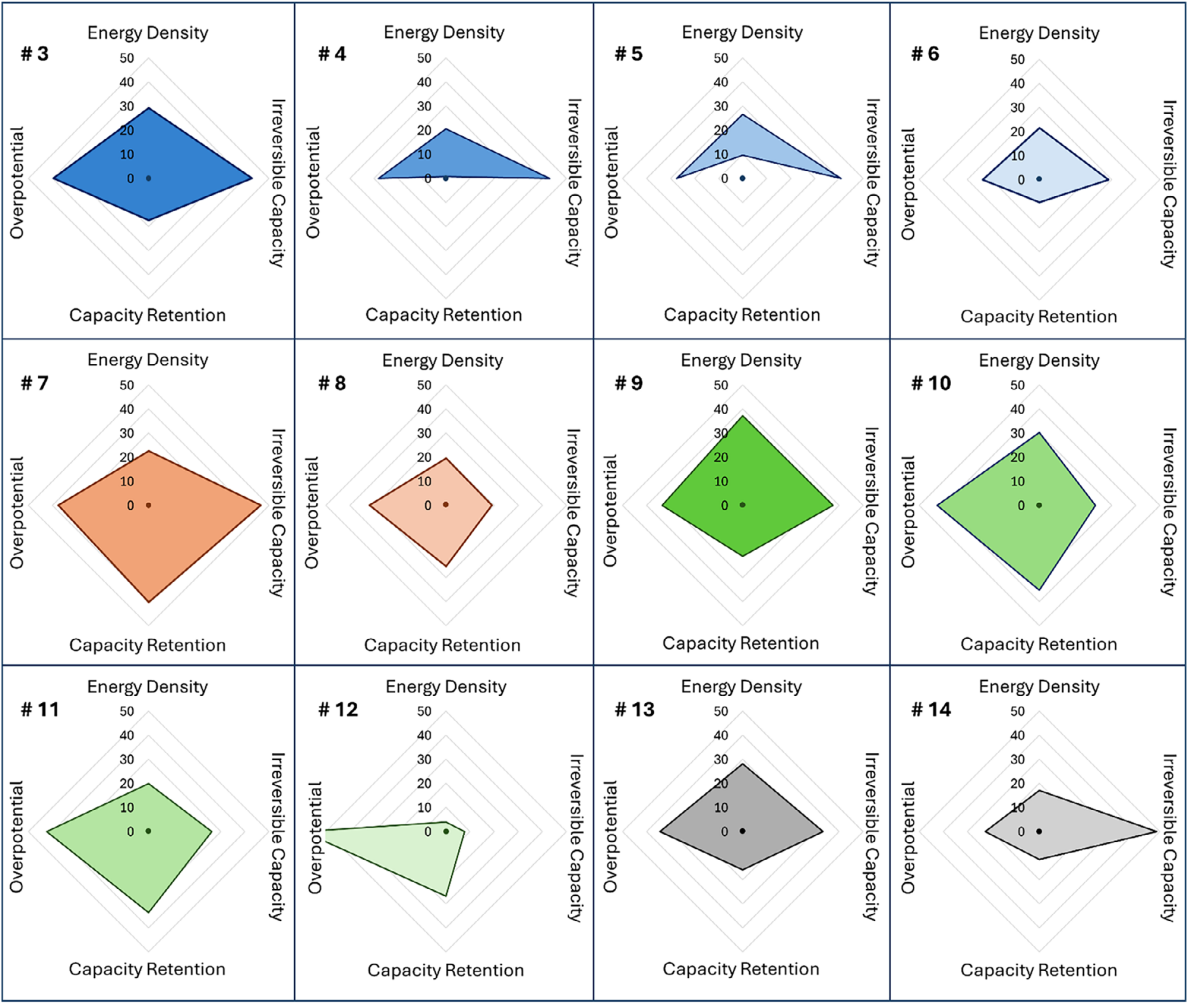
Radar plots representing percent improvements over the undoped material for each metric. Only the highest‐ranked (based on the FOM) materials from each round of active learning are shown.

The radar plot representations in Figure 4 provide an intuitive illustration of these results. The best composition from the 1st round of predictions (sample #7) is the material that shows the best balance between all metrics. By contrast, the best material from the 2nd round of predictions shows the very highest energy density, but at the cost of capacity retention. Similarly, the 1st round of predictions gave a material with the very best capacity retention but at the detriment of the energy density. These results demonstrate that the ML algorithms managed to navigate the well‐known trade‐offs in battery performance (it is common knowledge that energy density will invariably trade off to a certain extent with capacity retention). As a result, the predictions that attempt to equally prioritize all four metrics end up with two families of optimal compositions: those that favor energy density and those that favor lifetime. In either case, the area within the radar plot is clearly increased dramatically via the rounds of active learning.

Chemical composition analysis from Table 1 and Figure 4 collectively show that a number of the best‐performing materials contain indium. In ref. [19], we found indium to be highly beneficial in order to increase the electronic conductivity, a very important bottleneck in the LCP materials. Our original dataset prior to this study, was therefore heavily biased toward indium, as we had essentially not found any other elements that were efficient at overcoming the conductivity to yield strong specific capacities. Interestingly, Table 1 contains 4 materials without indium, and these are amongst the best performing. In fact, the top two random compositions did not contain indium, as did the second‐ranking material in the final set of predictions. On average, the active learning loop learns to favor chromium, niobium, scandium, lutetium, and phosphorus (10–30 % increase relative to the training distribution) while de‐emphasizing zinc, indium, and rhenium (6–17 % decrease, see Figure S5). The average stoichiometry of sampled dopants increases over the prior studies, and is similar to that of random samples (Figure S4). These analyses clearly demonstrate the important role that ML can play in diversifying the compositions of interest and overcome certain biases from the researchers. This becomes particularly significant in battery materials research, where materials abundance is an increasing concern, and the ability to diversify the list of elements used in our materials becomes a very important asset to large‐scale applications with an emphasis on sustainability.

**FIGURE 5:**
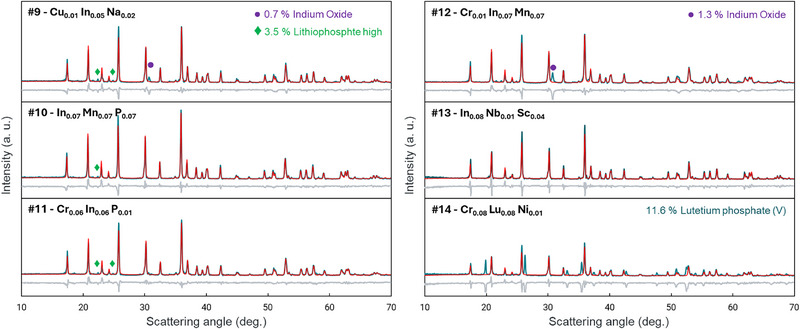
XRD of selected materials made in the last two rounds of active learning. The fits shown are single‐phase fits for the LCP phase, such that the unfit peaks are indications of the extent of secondary phases present. For each panel, the red line represents the calculated pattern, the green line represents the data, and the gray line is the residual.

It is also highly significant that the progress shown in improving the LCP material was based *solely* on battery performance metrics. The algorithms used to predict what materials to be made next were given the four battery performance metrics and the dopant composition only. No structural information was considered whatsoever. To garner some understanding as to what materials, in fact, were made, we performed X‐ray diffraction on certain materials from the last two rounds of predictions. The XRD patterns for those materials from Table 1 are shown in Figure 5. It is well known that for cathode materials, it is generally advantageous to have single‐phase materials in order to maximize most performance metrics (certainly the 3 metrics that are based on the first cycle, though synergy between two phases can improve extended cycling). It is therefore very interesting to see that most of the patterns in Figure 4 are very nearly single‐phase, even though no efforts were made to promote this during composition selection. The only exception to this is sample #14, which is also one of the samples that contains no indium. In this material, multiphase Rietveld fitting shows 11.6% of an electrochemically inactive secondary phase. This indicates that should we be able to make the pure phase material, the energy density should be considerably higher. These results are therefore highly informative in guiding future research and illustrate the power of this data‐driven approach to design new materials. All XRD patterns are included in the dataset provided.

We further analyzed the patterns by performing automated phase quantification using Rietveld refinement as detailed in ref. [19]. The results shown in Figure S13 show that generally a high LCP phase fraction above 85 % is obtained for the materials with the highest FOM, though no clear correlation is seen, highlighting the fact that numerous other parameters also come into play to yield high FOM. Ultimately, high LCP phase content is necessary for good battery performance, but not sufficient. In past work, we found certain dopants greatly suppressed the Li/Co antisite defects, and this led to improved extended cycling [19]. The current dataset undoubtedly also shows improved materials that come about through defect tuning. However, given that the XRD patterns show multiphase materials, we make no attempt here to quantify the defects, as this would be highly skewed by the variable compositions of the secondary phases. We leave it, therefore, as important follow‐up work to explore how defect engineering contributes toward a high FOM.

The final sample that warrants discussion is sample #12. This material shows quite moderate performance in all of our metrics, except the overpotential, which is by far the smallest obtained in our study (0.29 V). It is, in fact, solely due to the overpotential that it ranked highly in our FOM‐based rankings and is included in Table 1. However, visual examination of the cyclic voltammetry (Figure S3) makes it immediately evident that the apparent low overpotential (calculated from the average charge and discharge voltages) is due to an anomalous extra peak in the CV during the charge; in fact, the separation between the charge and discharge peaks in the CV is very comparable to all other samples. This is a clear example where having a human‐in‐the‐loop is highly beneficial. Thus, although our study overall shows the power of the ML‐driven closed‐loop approach, this particular sample illustrates the importance of having a human researcher involved in data analysis at some point: it is immediately dismissed as a misleading hit rather than a viable candidate.

## 4 Conclusions

Semi‐automated high‐throughput experiments have been highly effective in accelerating the design of battery components. However, there is a limit to the composition complexity that can be explored using traditional intuition‐based search methods. Herein, using chemical composition alone, we studied a machine‐learning approach to search through a composition space made up of about 15 million candidates. This is done with slightly less than a thousand experimental samples, and the objective is to co‐optimize 4 different battery metrics. Impressively, within 1 round of active learning (involving a batch of 64 predictions), a material was obtained that showed very strong improvement in all 4 metrics, and a number of other very strong candidates as well. Over the rounds of active learning, we see consistent improvements over average FOM, hypervolume, and hit rate, although there remains some poorly performing materials. This, and the fact that the approach was successful in generating beyond‐state‐of‐the‐art materials, highlights that ML can be highly effective without always being correct: the fact that our experimental workflow allows the simultaneous testing of 64 materials means that the benefits of ML become evident even though predictions are not all fully accurate. Our results also show that the ML‐guided approach is more reliable than random searching, where future work will study the use of more expressive featurization for improved generalization and other acquisition functions for faster convergence of the Pareto set, especially in larger search spaces. The closed‐loop approach showed a varied set of candidates in terms of elements and concentrations utilized, such that these methods are expected to be highly effective in diversifying elemental choices and lead toward more sustainable chemistries, an issue of growing importance as the scale of battery use grows dramatically.

## Conflicts of Interest

The authors declare no conflicts of interest.

## Supporting information




**Supporting File 1**: adma72533‐sup‐0001‐SuppMat.pdf.

**Supporting File 2**: adma72533‐sup‐0002‐Table‐ data‐LCP‐Advanced‐Materials.xlsx.

## Data Availability

The data that support the findings of this study are available in the supplementary material of this article. The code associated with the paper can be found at https://github.com/pchliu/BatteryBO.
