# Supplementary material for: Navigating Ternary Doping in Li‐ion Cathodes With Closed‐Loop Multi‐Objective Bayesian Optimization
Source: Adv Mater. 2026 Feb 12;38(16):e19790. doi: 10.1002/adma.202519790 (PMC12994333; doi:10.1002/adma.202519790)
Supplement: Supplementary file 1 — Supporting File 1: adma72533‐sup‐0001‐SuppMat.pdf. [file ADMA-38-e19790-s001.pdf]

SUPPORTING INFORMATION FOR

Navigating ternary doping in Li-ion cathodes with closed-loop multi-objective Bayesian optimization

Nooshin Zeinali Galabi[1,†], Cheng-Hao Liu [1,2,†,*] Moksh Jain[2,3], Marc Kamel[1], Shipeng Jia[1], Yoshua Bengio[2,3] and Eric McCalla[1,*]

1. McGill University, 801 Sherbrooke St. W, Montreal, Quebec, H3A 0B8, Canada.

2. Mila-Quebec AI Institute, 6666 Rue Saint-Urbain, Montreal, Quebec, H2S 3H1, Canada

3. Université de Montréal, 3150 Jean Brillant St, Montreal, Quebec, H3T 1N8, Canada

*e-mail: chenghao.liu@mail.mcgill.ca , eric.mccalla@mcgill.ca

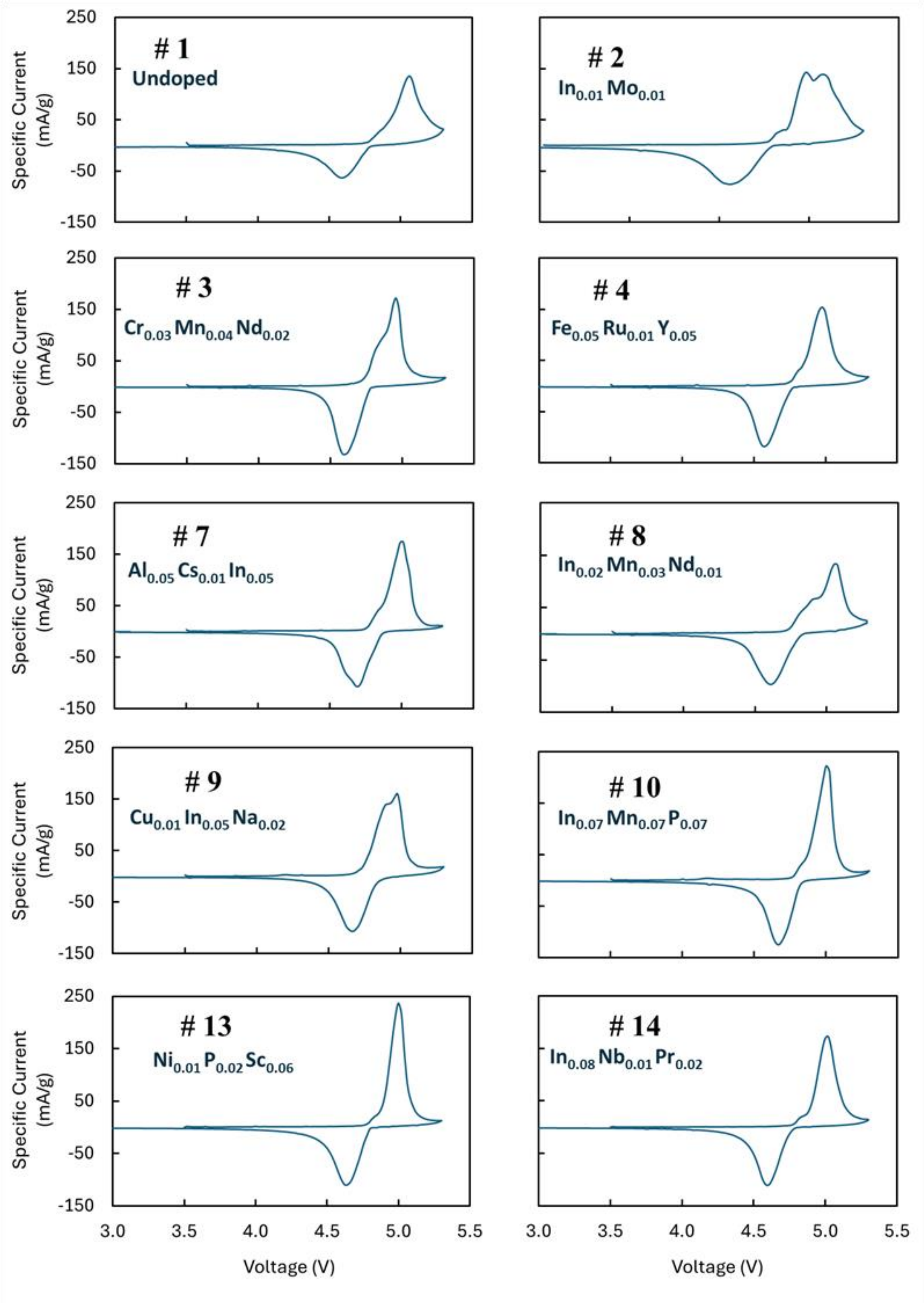

Figure S1: First cycle CVs.

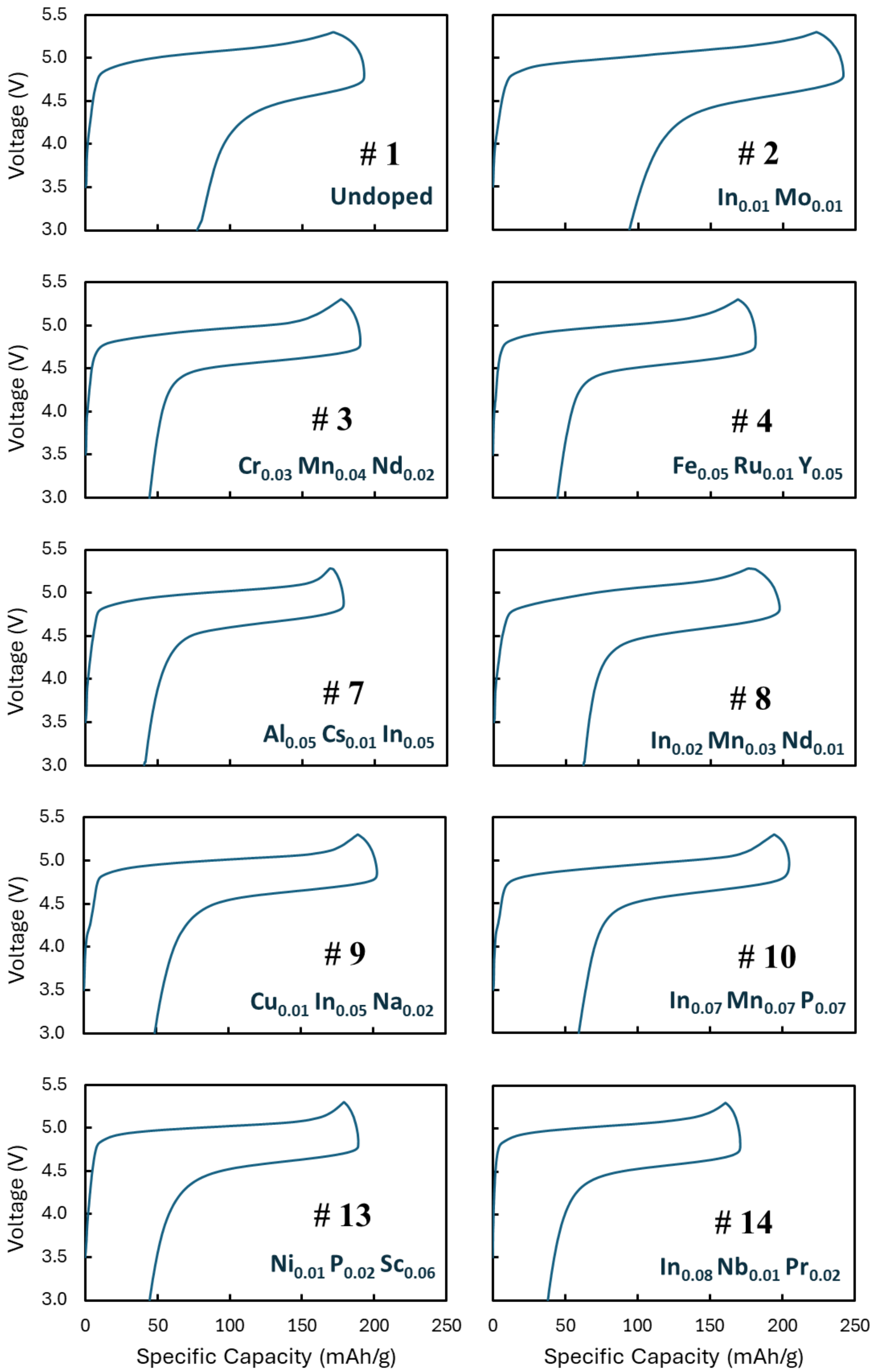

Figure S2: First cycle voltage curve for selected materials made herein. Voltage curves are calculated from the CVs.

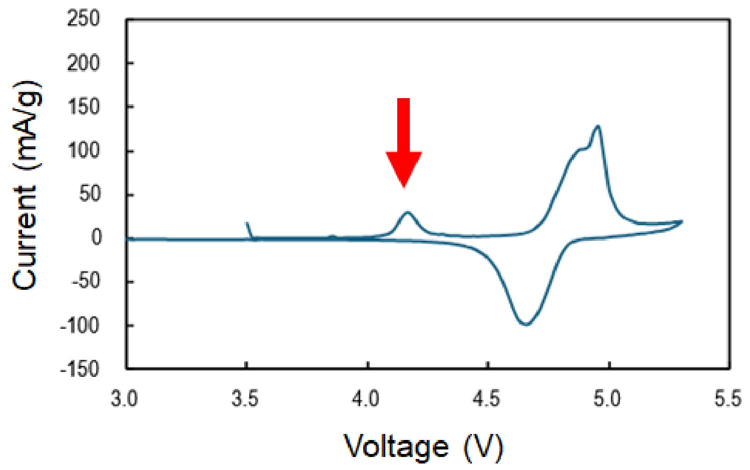

Figure S3: CV of sample #12 which yielded a particularly low overpotential with our automated data analysis. The peak highlighted with the red arrow is not seen in other samples and this is the root cause of the apparent lower overpotential. This is extremely easily identified visually, it is not straightforward to predict such outliers and ensure they are omitted.

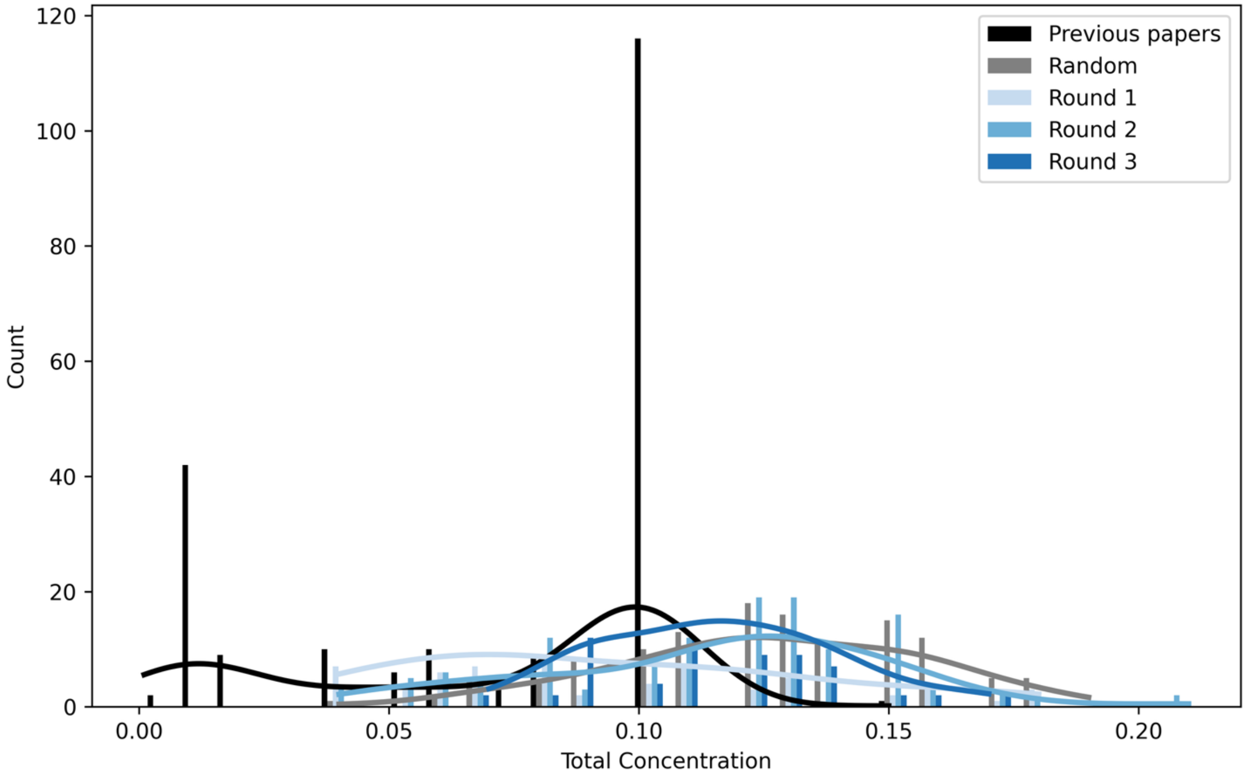

Figure S4. Distribution of the sum of dopant concentrations in previous papers, in randomly sampled tridopants, and in each round of active learning. The tridopants regularly sample beyond 10% dopant concentration, relative to cobalt.

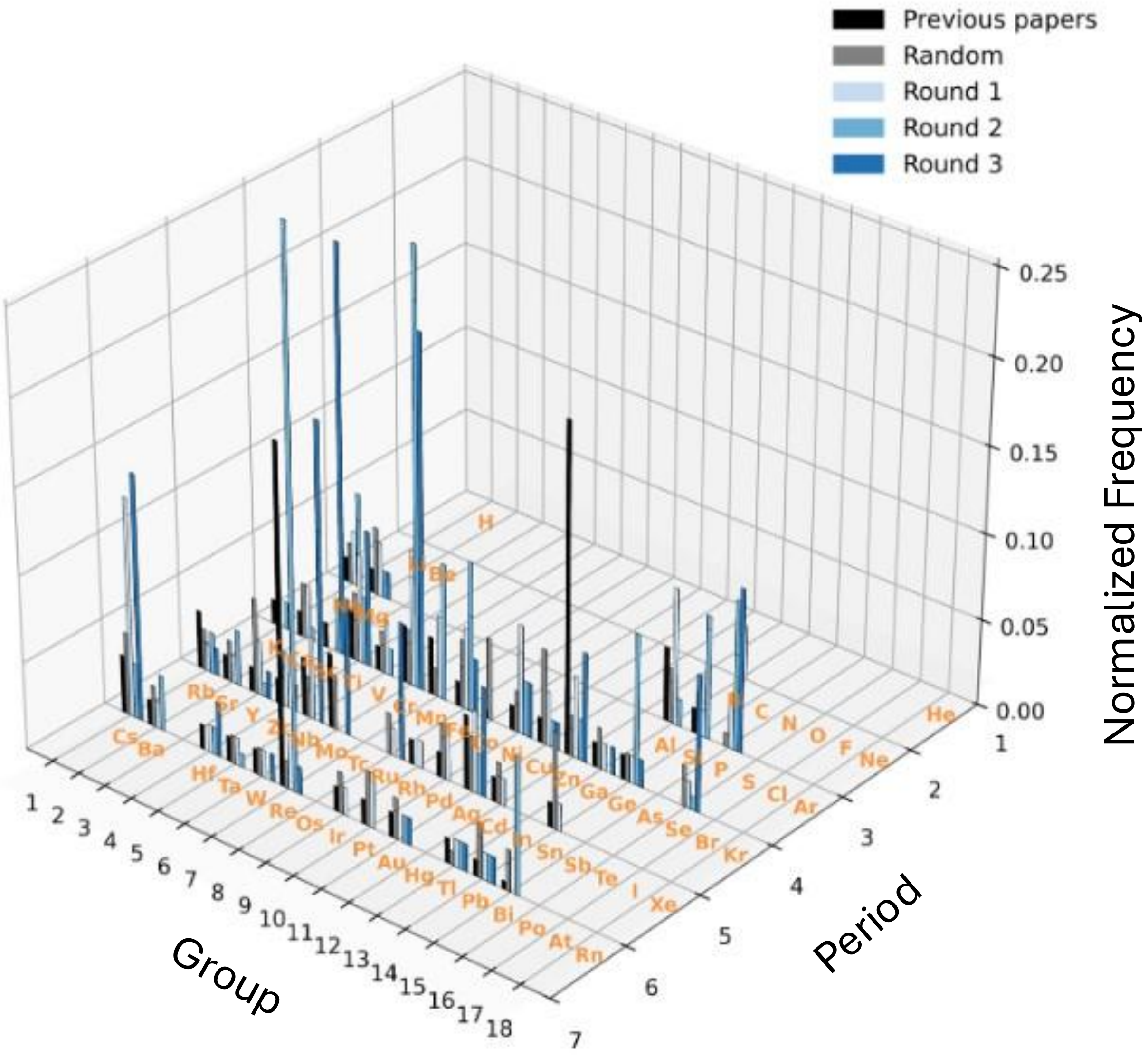

Figure S5. Distribution of elemental frequency of dopants in previous papers, in randomly sampled tridopants, and in each round of active learning. We removed In as in previous papers, we intentionally investigated many co-doped samples with indium, which biases its apparent frequency.

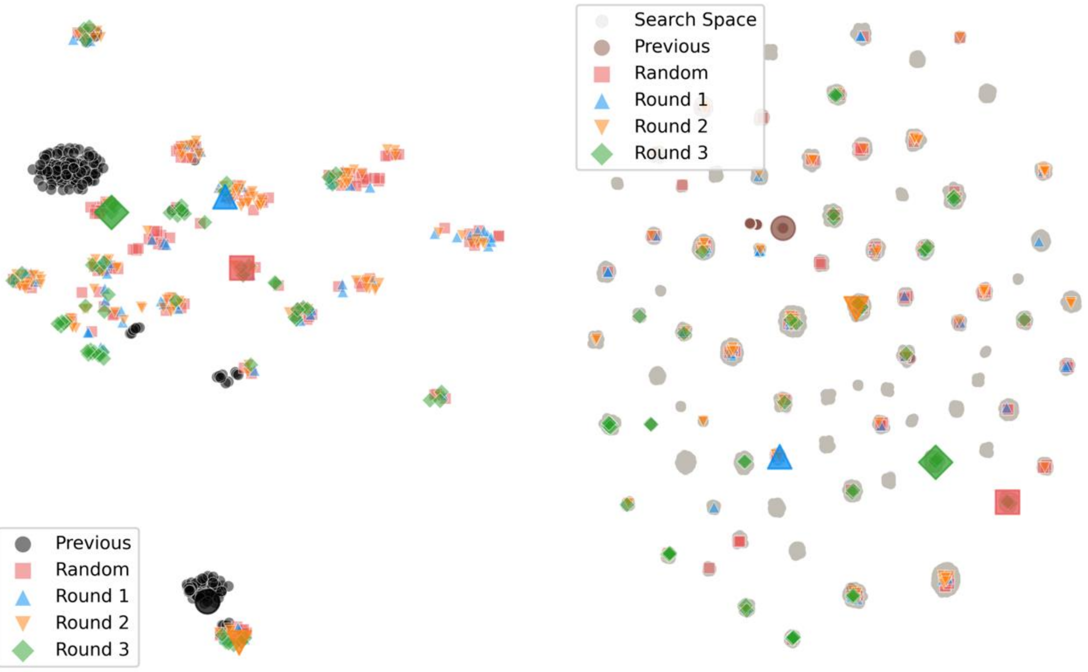

Figure S6. UMAP projected MatBERT composition embedding (num_neighbors = 100, min_dist = 0.75) of each round of candidates. Larger marker indicates the best sample in each dataset. (left) shows fit transform of just previous data, 126 random tridopant experiments, and the active learning rounds. (right) shows fit transform including 250,000 subsampled search space on top of all existing data.

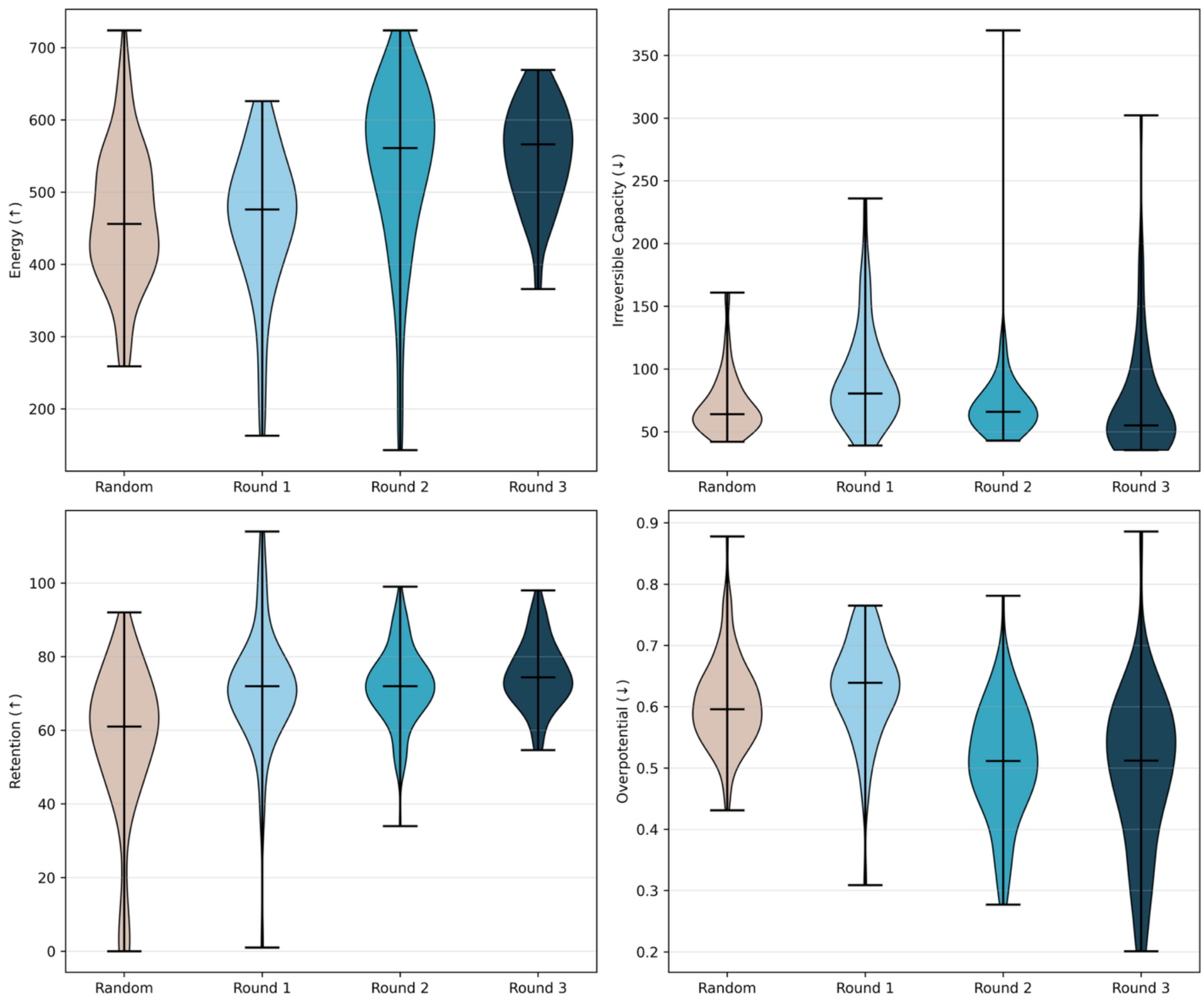

Figure S7. Violin plot of individual metrics across the random and active learning rounds.

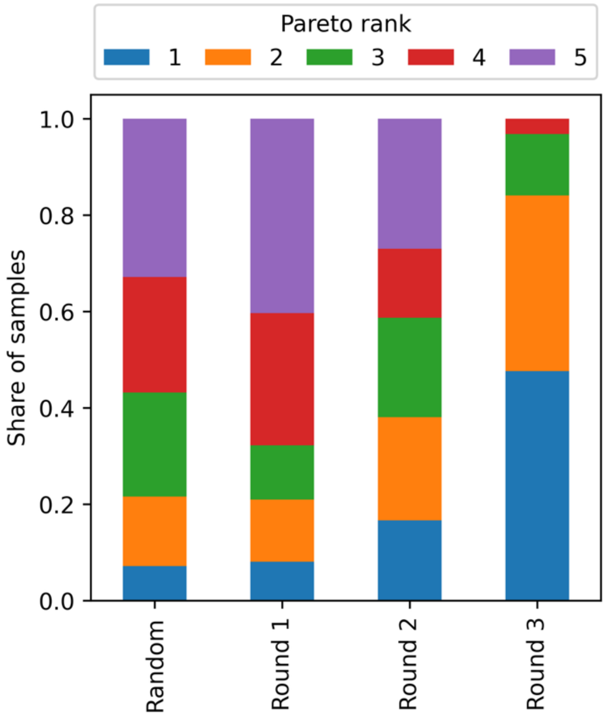

Figure S8. Pareto-depth distribution. Within each round, samples are compared on all objectives. Rank 1 means a sample is not worse on any objective and strictly better on at least one compared with any other sample; Rank 2 is the next front after removing Rank 1, and so on (capped at Rank = 5). More Rank 1–2 indicates better Pareto exploration and multi-objective performance.

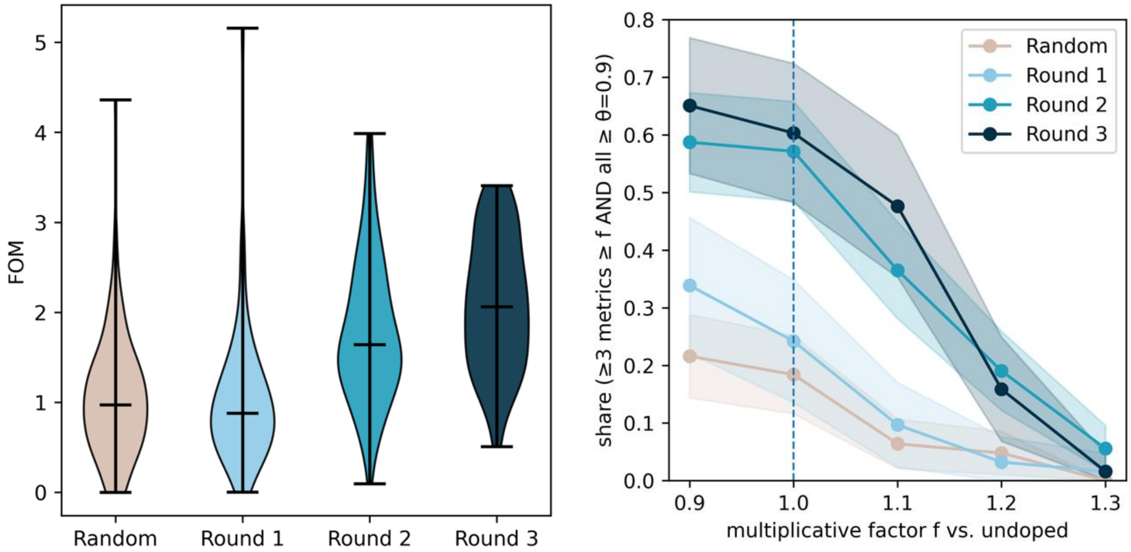

Figure S9. (Left) Violin plots of FOM across rounds, showing max and min. (Right) Simultaneous improvement compared to the undoped LCP. For each round, the curve shows the fraction of samples that is *f* times better than LCP on at least 3 of 4 metrics, while all 4 metrics also clear a baseline floor (here, 0.90). Higher curves indicate stronger *joint* improvement across metrics.

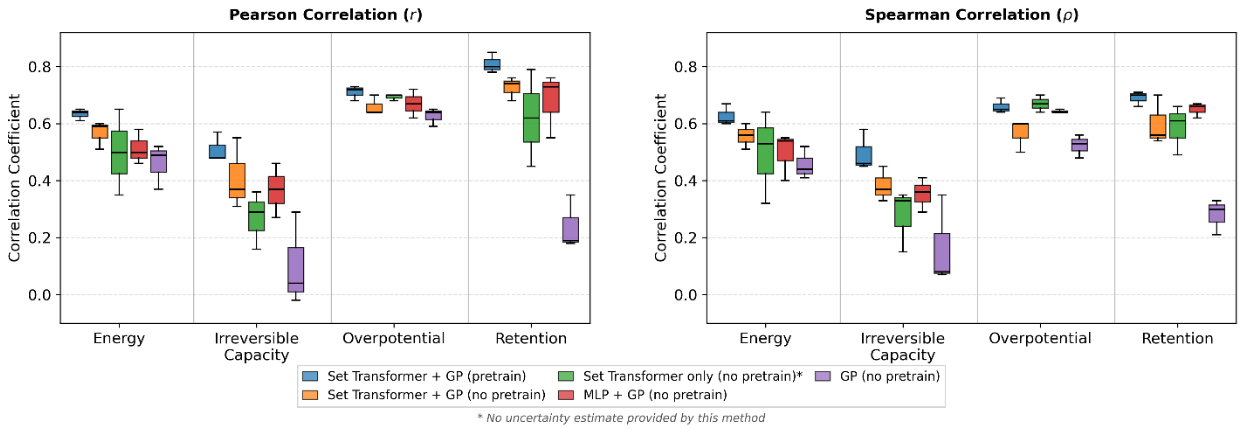

Figure S10. Validation correlation metrics comparing different ML models across four electrochemical performance metrics. "Pretrain" indicates models initialized with weights from DFT Fermi level regression; "no pretrain" indicates random initialization. All models were trained directly on the full experimental dataset without intermediate fine-tuning stage. Box plots show variation across 3 independent random seeds.

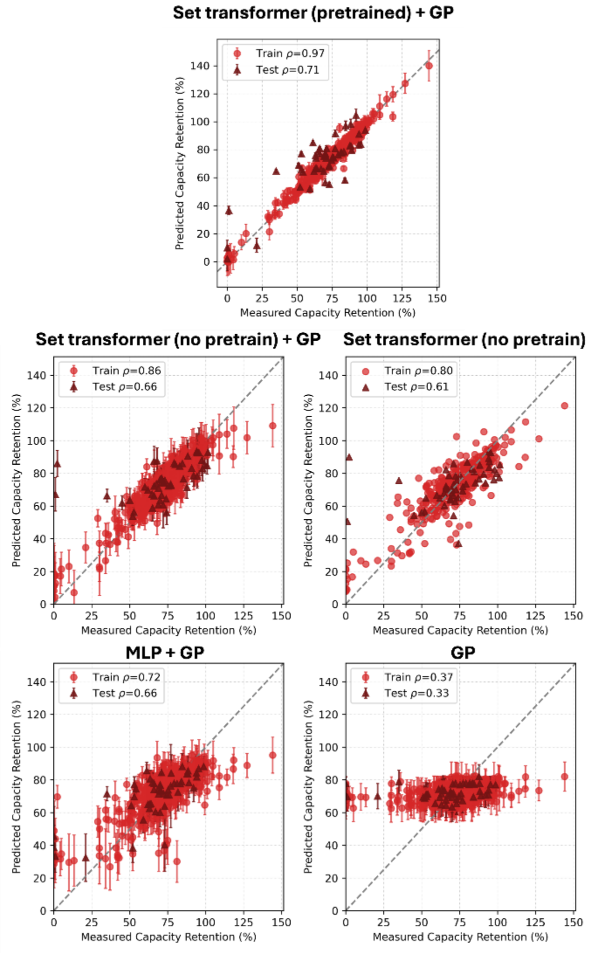

Figure S11. Example scatter plots of training and validation samples using different ML models.

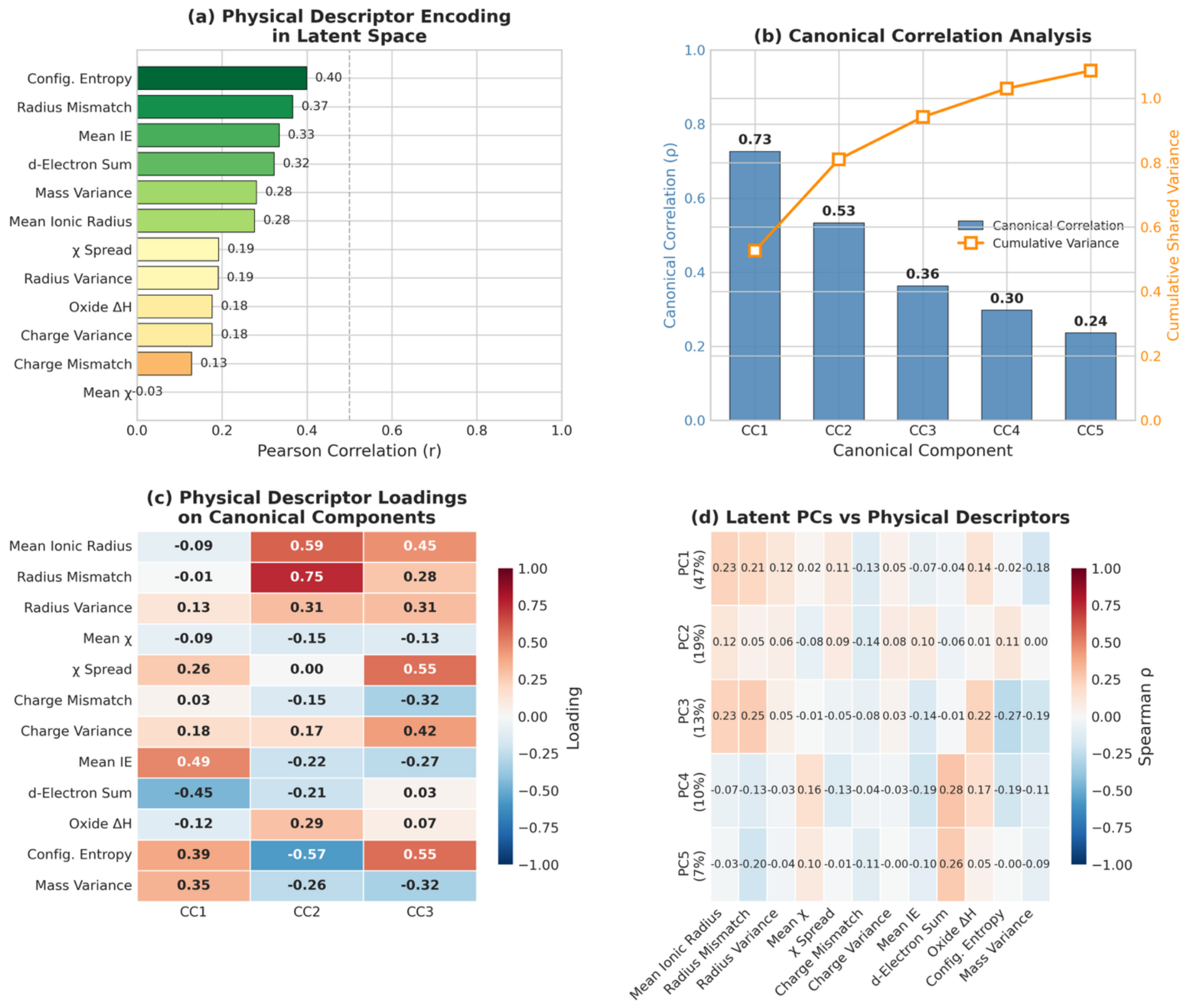

Figure S12. Physical interpretability of the deep kernel latent space. Physically motivated descriptors were computed directly from dopant identities and concentrations (ionic-size statistics such as mismatch relative to Co^{2+}, electronegativity statistics, oxidation/charge mismatch relative to Co^{2+}, ionization energy, d-electron count, oxide enthalpy proxy, configurational entropy, and mass variance) and compared against the set-transformer latent embeddings learned by the deep-kernel GP surrogate. **(a)** Linear-probe performance (Pearson *r* between predicted and true descriptor values) quantifies which physical descriptors are most recoverable from the latent space. **(b)** Canonical correlation analysis (CCA) identifies shared low-dimensional structure between learnt latent and physical spaces (bars: canonical correlations; line: cumulative shared variance proxy, $\Sigma\rho^2$). **(c)** Physical-descriptor loadings on the first three canonical components highlight which descriptors dominate each shared mode. **(d)** Spearman correlations between principal axes of the latent space and physical descriptors provide an additional, axis-wise view of alignment. The distributed nature of correlations across PCs indicates that physical information is encoded in a non-axis-aligned manner.

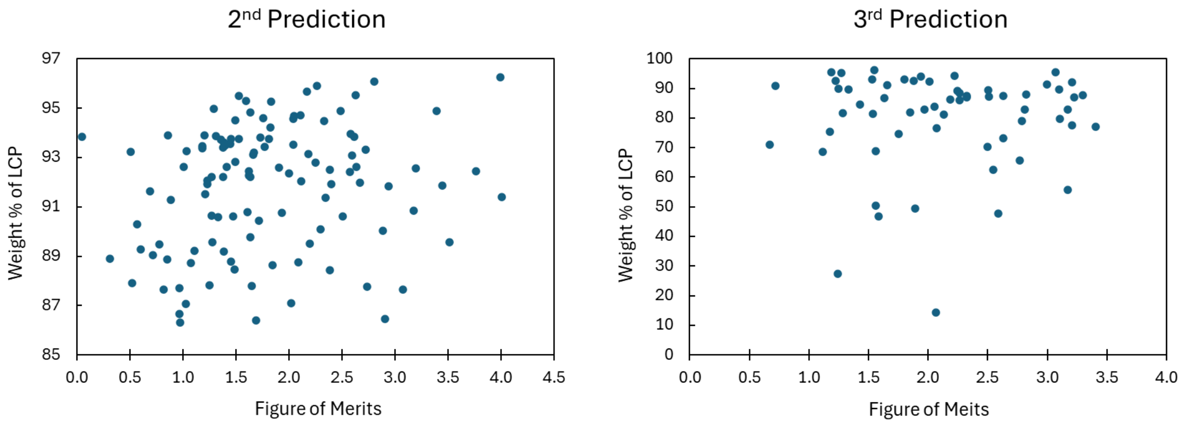

Figure S13. Plots of the percentage of LCP phase based on XRD Rietveld fitting vs. the FOM for all 186 samples from the last two rounds of predictions.
